# Antimicrobial, antimycobacterial and antibiofilm properties of *Couroupita guianensis* Aubl. fruit extract

**DOI:** 10.1186/1472-6882-12-242

**Published:** 2012-12-04

**Authors:** Naif Abdullah Al-Dhabi, Chandrasekar Balachandran, Michael Karunai Raj, Veeramuthu Duraipandiyan, Chinnasamy Muthukumar, Savarimuthu Ignacimuthu, Inshad Ali Khan, Vikrant Singh Rajput

**Affiliations:** 1 Department of Botany and Microbiology, Addiriyah Chair for Environmental Studies, College of Science, King Saud University, P.O. Box 2455, Riyadh, 11451, Saudi Arabia; 2 Division of Microbiology, Entomology Research Institute, Loyola College, Chennai, 600 034, India; 3 Research and Development Centre, Orchid Chemicals and Pharmaceuticals Ltd, Sozhanganallur, Chennai, 600119, India; 4 Clinical Microbiology Division, Indian Institute of Integrative Medicine, Jammu, 180 001, India

**Keywords:** Antimicrobial, Antimycobacterial, Antibiofilm, *Couroupita guianensis*, Indirubin

## Abstract

**Background:**

*Couroupita guianensis* Aubl. (Lecythidaceae) is commonly called Ayahuma and the Cannonball tree. It is distributed in the tropical regions of northern South America and Southern Caribbean. It has several medicinal properties. It is used to treat hypertension, tumours, pain, inflammatory processes, cold, stomach ache, skin diseases, malaria, wounds and toothache.

**Methods:**

The fruits of *Couroupita guianensis* were extracted with chloroform. Antimicrobial, antimycobacterial and antibiofilm forming activities of the chloroform extract were investigated. Quantitative estimation of Indirubin, one of the major constituent, was identified by HPLC.

**Results:**

Chloroform extract showed good antimicrobial and antibiofilm forming activities; however it showed low antimycobacterial activity. The zones of inhibition by chloroform extract ranged from 0 to 26 mm. Chloroform extract showed effective antibiofilm activity against *Pseudomonas aeruginosa* starting from 2 mg/mL BIC, with 52% inhibition of biofilm formation. When the chloroform extract was subjected to HPLC-DAD analysis, along with Indirubin standard, in the same chromatographic conditions, it was found that Indirubin was one of the major compounds in this plant (0.0918% dry weight basis).

**Conclusions:**

The chloroform extract showed good antimicrobial and antibiofilm properties. Chloroform extract can be evaluated further in drug development programmes.

## Background

*Couroupita guianensis* Aubl. (Lecythidaceae) is commonly called Ayahuma and the Cannonball tree. It is an evergreen tree allied to the Brazil Nut (*Bertholletia excelsa*) and is native to tropical northern South America and the Southern Caribbean [1]. The trees are grown extensively in Shiva temples in India. Hindus revere it as a sacred tree because the petals of the flower resemble the hood of the Naga, a sacred snake, protecting a Shiva Lingam, the stigma. The tree also produces globular brown woody, indehiscent, amphisarcun (double fleshy) fruits of an astonishing size, almost the size of a human head [2]. It is widely planted in tropical and subtropical botanical gardens as an ornamental; it does well under cultivations and it is used to feed animals. Native Amazonian people from Amazonian region and other states of the north region of Brazil use infusions or teas obtained from the leaves, flowers, and barks of *Couroupita guianensis* to treat hypertension, tumours, pain, and inflammatory processes [3]. The Cannonball tree possesses antibiotic, antifungal, antiseptic and analgesic qualities. The trees are used to cure cold and stomach ache. Juice made from the leaves is used to cure skin diseases, and shamans of South America have even used tree parts for treating malaria. The inside of the fruit can disinfect wounds and young leaves cure toothache [4]. Chemical studies of this species showed the presence of α-amirin, β-amirin, β-sitosterol, nerol, tryptanthrine, indigo, indirubin, isatin, linoleic acid, carotenoids and sterols [5-10]. In the flowers, it was possible to identify eugenol, linalool and (E,E)-farnesol where as triterpenoid esters of fatty acids as β-amirin palmitate were characterized in the leaves [11]. Indirubin is a purple 3,2′bisindole, and is a constituent of indigo natural. Indigo natural is a dark blue powder prepared from the leaves of a number of medicinal plants including *Baphicacanthus cusia* (Acanthaceae), *Polygonum tinctorium* (Polygonaceae), *Isatis indigotica* (Brassicaceae), *Indigofera suffrutticosa* (Fabaceae) and *Indigofera tinctoria* (Fabaceae) [12]. Indigo, naturally is used in traditional Chinese medicine as a hemostatic, antipyretic, antiinflammatory, and sedative agent in the treatment of bacterial and viral infections [13]. In the present communication we report the antimicrobial, antimycobacterial and antibiofilm forming activities of the chloroform extract of the fruit of *C*. *guianensis*. The HPLC finger print of the chloroform extract together with quantification of Indirubin as marker is also given.

## Materials and methods

### Plant material

Fresh fruits of *Couroupita guianensis* were collected during June 2011 from Loyola College Jesuit garden, Chennai, India. The species was identified by a plant taxonomist at Entomology Research Institute, Loyola College, Chennai, India. A voucher specimen (No. ERI/ETHPH/CQ/225) was deposited at the herbarium of the institute.

### Preparation of plant extract

The collected fruit was shade dried at room temperature and powdered. 1 kg of fruit powder was extracted with chloroform at room temperature for 48 hrs. The extract was evaporated to dryness at 40°C under reduced pressure.

### Microbial organisms

The following Gram positive, Gram negative bacteria, clinical isolates and fungi were used for the experiment.

### Gram positive bacteria

*Bacillus subtilis* MTCC 441, *Micrococcus luteus* MTCC 106, *Enterobacter aerogenes* MTCC 111 and *Staphylococcus aureus* MTCC 96.

### Gram negative bacteria

*Shigella flexneri* MTCC 1457, *Salmonella paratyphi*-*B*, *Klebsiella pneumoniae* MTCC 109, *Pseudomonas aeruginosa* MTCC 741, *Proteus vulgaris* MTCC 1771 and *Salmonella typhimurium* MTCC 1251.

### Clinical isolates

*Escherichia coli* (ESBL-3984,), *Escherichia coli* (ESBL-3904), *Klebsiella pneumoniae* (ESBL-3971), *Klebsiella pneumoniae* (ESBL-75799), *Klebsiella pneumoniae* (ESBL-3894), *Klebsiella pneumoniae* (ESBL-3967) and *Staphylococcus aureus* (MRSA).

### Fungi

*Candida albicans MTCC 227* and *Malassesia pachydermatis*; The reference cultures were obtained from Institute of Microbial Technology (IMTECH), Chandigarh, India-160 036 and Department of Microbiology, Christian Medical College, Vellore, Tamil Nadu, India.

### Preparation of inoculum

Bacterial inoculums were prepared by growing cells in Mueller Hinton Broth (MHB) (Himedia, Mumbai) for 24 hrs at 37°C. Yeast was grown on Sabouraud Dextrose Broth (SDB) (Himedia, Mumbai) at 28°C for 48 hrs.

### Antibacterial activity

Antibacterial activity was carried out using disc diffusion method [14]. Petri plates were prepared with 20 mL of sterile Mueller Hinton Agar (MHA). The test cultures were swabbed on the top of the solidified media and allowed to dry for 10 min. A specific concentration (5 mg/disc) of the chloroform extract was loaded to each disc. The loaded discs were placed on the surface of the medium. Negative control was prepared using respective solvents. Streptomycin was used as positive control. The plates were incubated for 24 hrs at 37°C for bacteria and for 48 hrs at 28°C for fungi. Zones of inhibition were recorded in millimetres and the experiment was repeated twice.

### Antimycobacterial assay

The anti-TB activity of chloroform extract was evaluated against standard sensitive strain *M*. *tuberculosis* H_37_Rv and rifampicin isolate *M*. *tuberculosis* XRD-1. The mycobacterial cultures were obtained from Clinical Microbiology Division, Indian Institute of Integrative Medicine, Jammu 180 001, India. The minimum inhibitory concentration (MIC) was determined using broth micro-dilution assay [15,16]. The experiment was performed in sterile Middlebrook 7H9 broth supplemented with 10% ADC (BD Biosciences, USA). The above-mentioned test bacteria were grown to mid-log phase (10–12 days) at 37°C with shaking in the test media (Middlebrook 7H9 broth supplemented with 10% ADC). Stock solution (1 mg/mL) of chloroform extract was prepared in DMSO and 6.4 μl volume of these stock solutions were added to the wells of a 96 well U bottom microtitre plates (Tarson, Mumbai, India) and nine 2 fold serial dilutions of the compound were prepared in 100 μl of test media. The turbidity of the cultures was adjusted to be equivalent to 1 McFarland turbidity standard (~1 x 10^7^ CFU/mL), which was further diluted to 1:10 in test media and a 100 μl volume of this diluted inoculum was added to each well of the plate, resulting in a final inoculum of 5 x 10^5^ CFU/mL. The final concentrations of the chloroform extract after the addition of inoculums ranged from 0.12 to 32 μg/mL. Rifampicin in the concentration range from 0.12 to 32 μg/mL was used as control drug in the experiment. Periphery wells of the plate were filled with sterile distilled water to prevent evaporation of media in the wells. The plates were incubated at 37°C under 5% CO_2_ for 3 weeks. Inhibition of growth was determined both by visual examination and with a spectrophotometer at an OD_600_ (Multiskan spectrum; Thermo Scientific, USA). The lowest concentration of the compound showing no turbidity was recorded as MIC.

### Effect of chloroform extract of *C*. *guianensis* on biofilm formation

The effect of the Chloroform extract on biofilm forming activity of *P*. *aueroginosa* was tested on 24-well polystyrene plates. Chloroform extract at concentrations of 1–5 mg/mL were added in LB containing the bacterial suspension at 10^6^ CFUmL/1. The plates were incubated for 24 h at 37°C. After incubation, biofilm was stained with 0.4% crystal violet. The biofilm inhibitory concentration (BIC) was determined as the lowest concentration that produced visible disruption in biofilm formation and significant reduction in the readings when compared with the control wells at OD570nm. Thus, the BIC was determined by both spectrophotometric quantification and also by microscopic visualization. For visualization of biofilms by light microscopy, the biofilms were allowed to grow on glass pieces (1/1 cm) placed in 24-well polystyrene plates supplemented with the extracts (1–5 mg/mL) and incubated for 24 h at 37°C. Crystal violet staining was performed as described above. Stained glass pieces were placed on slides with the biofilm pointing up and were inspected by light microscopy at magnifications of X40. Visible biofilms were documented with an attached digital camera (Nikon Eclipse Ti 100) [17].

### Standardization of chloroform extract by HPLC-DAD analysis

Sample (chloroform extract) (10 mg in 100 mL) and standard Indirubin (5 mg in 100 mL) were dissolved in methanol. The solutions were filtered through a membrane filter (pore size 0.20 μm) prior to HPLC analysis.

HPLC analysis was carried out on a Waters Alliance 2695 separations Module with photodiode array detector (Waters, 2996). The LC column was an YMC pack ODS A (150 mm × 4.6 mm, 5 μm) column. Two mobile phases A and B were used at flow rate of 1.0 mL/min. The mobile phase was filtered through a 0.45 μm filter, and degassed by vacuum, followed by sonication. Mobile phase A consisted of water with 0.1% orthophosphoric acid and mobile phase was B acetonitrile. Separation was carried out at room temperature. A gradient was used, starting at 95% A, changing to 10% A linearly in 15 min. After elution the column was re-equilibrated for 3 min under the initial conditions. The HPLC profile of *C*. *guianensis* chloroform extract was compared with that of standard compound, Indirubin which was best detected at 254 nm.

### Statistics

Statistical analysis was performed using SPSS. Values were expressed as mean ± SD. A Duncan–ANOVA test was used to compare parameters between groups and a Dunnett–ANOVA test to compare between tests and control.

## Results and discussion

**Figure 1:**
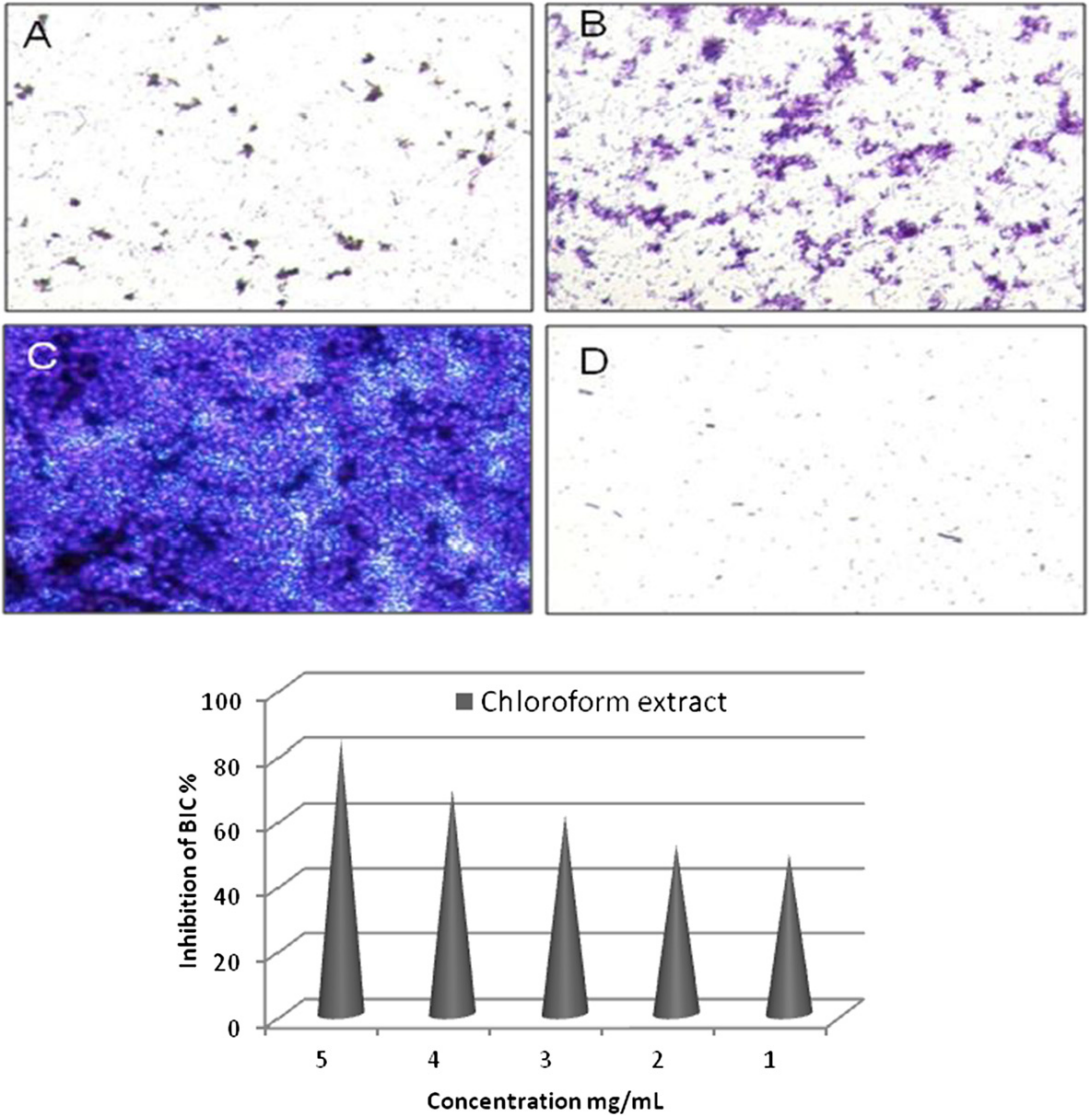
**Microscopic visualization** (**x40**) **of antibiofilm activity of low concentration of chloroform extract on *Pseudomonas aeruginosa***: (**A**) **2**.**0 mg**/**mL**, (**B**) **1**.**0 mg**/**mL**, (**C**) **control and** (**D**) **Negative control** (**Indirubin**).

In this communication we report the antimicrobial, antimycobacterial and antibiofilm forming activities of the chloroform extract of *C*. *guianensis* fruit. Chloroform extract of *C*. *guianensis* exhibited promising activity against bacteria and fungi using disc diffusion method. The activity of chloroform extract against bacteria and fungi are given in Table 1. The compound showed appreciable activity against Gram positive bacteria, *B*. *subtilis* (14 mm), *M*. *luteus* (18 mm), *E*. *aerogenes* (19 mm) and *S*. *aureus* (26 mm); Gram negative bacteria *S*. *flexneri* (20 mm), *K*. *pneumonia* (18 mm), *P*. *aeruginosa* (8 mm) and *P*. *vulgaris* (12 mm); Clinical isolates ESBL-3984 (20 mm), ESBL-3904 (18 mm), ESBL-3971 (14 mm), ESBL-75799 (16 mm), ESBL-3894 (15 mm), ESBL-3967 (12 mm) and MRSA (18 mm); Fungi *C*. *albicans* (8 mm) and *M*. *pachydermatis* (16 mm). Compared to control, the chloroform extract of *C*. *guianensis* showed moderate activity against tested bacteria and fungi. *C*. *guianensis* fruit was previously shown to have good *in vitro* antibacterial activity in few human pathogens [18,19]. Hence in this study we gave more importance to clinical isolates. *C*. *albicans* which causes candidiasis is becoming an increasingly important species worldwide, due to the fact that it is among the opportunistic pathogens frequently found in AIDS patients [20]. The chloroform extract of *C*. *guianensis* showed good activity against *C*. *albicans*. The chloroform extract was inactive against two strains of *Mycobacterium tuberculosis* at tested concentration of 64 μg/mL, thus showing low activity as compared with rifampicin (Table 2). As shown in Figure 1 and Table 3, the chloroform extract inhibited biofilm formation against *P*. *aueroginosa* starting from 2.0 mg/mL (BIC). Interestingly, the chloroform extract showed a pronounced effect on inhibition of biofilm formation at low concentrations. The chloroform extract showed effective antibiofilm formation activity at 2.0 mg/mL (BIC), with 52% inhibition. The efficiency of the extract was also confirmed by microscopic visualization. The antibiofilm formation activity was low at higher concentration. This indicated that the biofilm formation was possibly inhibited at the beginning of the attachment stage.

**Table 1:** **Antimicrobial activity of the chloroform extract of *C***. ***guianensis *fruits** (**5 mg**/**mL**)

**Organism**	**Chloroform extract**	**Streptomycin**
**Gram positive**		
*S*. *aureus*	26	14
*E*. *aerogens*	19	22
*M*. *luteus*	18	26
*B*. *subtilis*	14	22
**Gram negative**		
*S*. *flexneri*	20	30
*P*. *vulgaris*	12	30
*S*. *paratyphi*-*B*	-	24
*S*. *typhimurium*	-	18
*P*. *aeruginosa*	8	30
*K*. *pneumonia*	18	20
**Clinical isolates**		
*E*. *coli* (ESBL-3984)	20	12
*E*. *coli* (ESBL-3904)	18	12
*K*. *pneumoniae* (ESBL-3971)	14	16
*K*. *pneumoniae* (ESBL-75799)	16	16
*K*. *pneumoniae* (ESBL-3894)	15	14
*K*. *pneumoniae* (ESBL-3967)	12	16
*S*. *aureus* (MRSA)	18	-
**Fungi**		Ketoconazole
*C*. *albicans*	8	28
*M*. *pachydermatis*	16	26

(−) no activity; Streptomycin (standard antibacterial agent); Ketoconazole (standard antifungal agent); Negative control (solvent) (Nil); N = 2.

**Table 2:** **Minimum inhibitory concentration of the chloroform extract of *C***. ***guianensis *against *M***. ***tuberculosis***

**Strains**	**Lab code**	**Chloroform extract MIC** (μ**g**/**mL**)	**Rifampicin** (μ**g**/**mL**)
*M*. *tuberculosis* H^37^Rv (HR-Sen) ATCC 27294	H_37_Rv	>64	0.12
*M*. *tuberculosis* XRD-1	XRD	>64	32

Negative control: Nil growth.

**Table 3:** **BIC of chloroform extract against the biofilm forming activity of *Pseudomonas aeruginosa *strain at 24 h**

**S**.**No**	**Chloroform extract**(**mg**/**ml**)	**Antibioflim activity**
1	5.0	0.546 ± 0.00145
2	4.0	0.581 ± 0.00176
3	3.0	0.637 ± 0.00644
4	2.0	0.712 ± 0.00318
5	1.0	0.941 ± 0.00260
6	Control	1.706 ± 0.00240

Effect of chloroform extract on biofilm formation of *Pseudomonas aeruginosa* strain as quantified by crystal violet staining and measuring A570nm. Mean values of triplicate independent experiments and SD are shown (N = 3) (Negative control-Nil).

The bioactive compounds present in the extracts might have interfered with the adherence of *P*. *aueroginosa* by releasing the adhesion compound lipoteichoic acid from the streptococcal cell surface [21]. *P*. *aeruginosa* has emerged as one of the most problematic Gram-negative pathogen, with an alarmingly high antibiotic resistance rate [22,23]. The activity of *C*. *guianensis* might be due to its ability to complex with cell wall [19] and thus inhibiting the microbial growth [24]. An important step in biofilm development is the formation of the characteristic biofilm architecture [25]. Cell surface charge and CSH play a crucial role in bacterium–host cell interactions [26]. There are several reports regarding plant extracts interfering in the biofilm formation of Gram-negative bacteria [27] and Gram-positive bacteria [28-30]. Biofilm-associated diseases caused by Gram-positive bacteria include caries, gingivitis, periodontitis, endocarditis and prostatitis [31]. Many forms of streptococcal infections, especially recurrent and chronic infections, are associated with the formation of bacterial biofilms [32]. This phenomenon is also observed in antibiotics where the sub-inhibitory concentrations (sub-MICs) of antibiotics, although not able to kill bacteria, can modify their physicochemical characteristics and the architecture of their outermost surface and may interfere with some bacterial functions.

**Figure 2:**
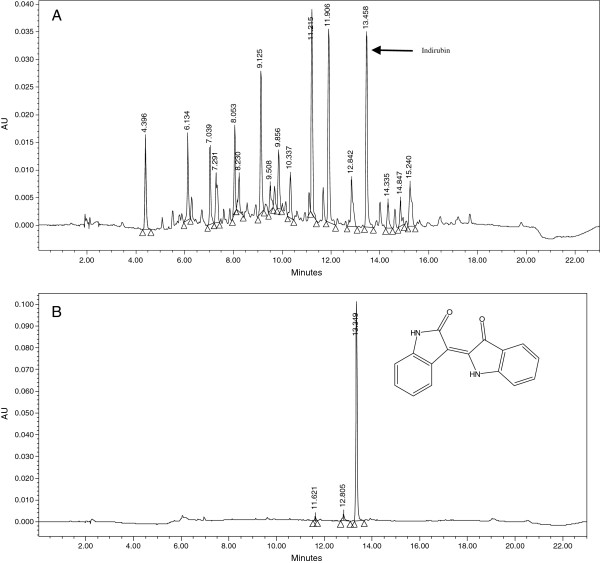
**HPLC chromatograms of the chloroform extract of the fruit of *Couroupita guianensis*** (**A**) **HPLC chromatogram of standard of Indirubin** (**B**). **Chloroform extract.** Indirubin.

**Figure 3:**
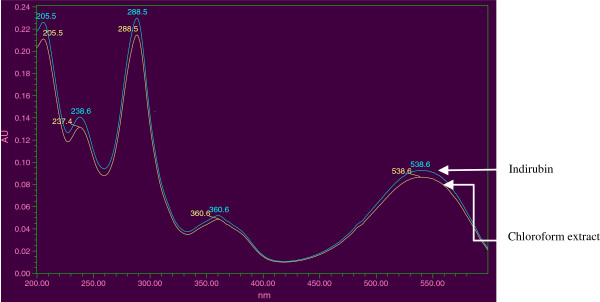
**UV spectrum overlay of Indirubin standard and the corresponding peak of chloroform extract of the fruit of *Couroupita guianensis.*** The retention time of the standard compound with UV detection at 254 nm was about 13.4 min. The UV spectral similarity was also confirmed by the overlay of the UV spectrum of the standard to the corresponding peak of chloroform extract of the fruit of *C*. *guianensis*, extracted from PDA detection.

When the chloroform extract was subjected to HPLC-DAD analysis, along with Indirubin standard, in the same chromatographic conditions, it was found that Indirubin was one of the major compounds in this plant. The retention time of the standard compound with UV detection at 254 nm was about 13.4 min. The UV spectral similarity was also confirmed by the overlay of the UV spectrum of the standard to the corresponding peak of chloroform extract of the fruit of *C*. *guianensis*, extracted from PDA detection. The HPLC chromatogram of the chloroform extract with that of standard Indirubin is given in Figure 2 and the overlay UV spectrum of the Indirubin standard to the corresponding peak of chloroform extract of the fruit of *C*. *guianensis* is given in Figure 3. The above HPLC quantification showed that the Indirubin content of the fruits of *C*. *guianensis* (dry weight basis) to be 0.0918%. Indirubin has been used as antibacterial and antifungal agent, particularly, to cure fungal diseases, dermatophytic and skin lesion diseases [33]. Indirubin, a natural purple pigment, occurs as 3, 20-bisindole; it has been shown to be active for the treatment of chronic myelocytic leukemia [13]. While parent indirubin molecule is derived from the non-enzymatic and spontaneous dimerization of colorless precursors, isatin and indoxyl, in the indigo-producing plants, a series of novel derivatives, have been synthesized by various molecular substitution of the parental indirubin backbone with improved solubility, selectivity and bioavailability [34-37]. Indirubin derivatives have been shown to inhibit cyclin-dependent kinases (CDKs), glycogen synthase kinase (GSK)-3 and activate aryl hydrocarbon receptor (AhR). Anticancer activity of indirubin in human cancer cells, such as MCF-7, HBL-100 breast cancer cells, HT-29 colon adenocarcinoma, haematopoietic cell lines Jurkat, and A498, CAKI-1, AKI-2 renal cancer cells [12,37-40] have been reported. Indirubin could also suppress autophosphorylation of fibroblast growth factor receptor (FGFR)-1 but stimulate extracellular signal regulated kinase (ERK1/2) activity through p38 mitogen-activated protein kinase [41].

## Conclusion

The chloroform extract of the fruit of *C*. *guianensis* showed good antimicrobial activities but low antimycobacterial activity against tested strains. The antibiofilm forming activity of the chloroform extract showed a pronounced effect on inhibition of biofilm formation at low concentrations with 52% inhibition.

## Competing interests

The authors declare that they have no competing interests.

## Authors’ contributions

NAA-D designed and supervised the *experimental* work and evaluated the data. CB, VD, CM and NAA-D carried out the study; MK carried out the HPLC analysis; SI supervised the work and corrected the manuscript; IAK and VSR carried out the antimycobacterial work. All authors have read and approved the final manuscript.

## Pre-publication history

The pre-publication history for this paper can be accessed here:

http://www.biomedcentral.com/1472-6882/12/242/prepub
